# Use of voriconazole to predict susceptibility and resistance to isavuconazole for *Aspergillus fumigatus* using CLSI methods and interpretive criteria

**DOI:** 10.1128/jcm.01207-24

**Published:** 2024-12-20

**Authors:** Marisa L. Winkler, Paul R. Rhomberg, Kelley A. Fedler, Michael D. Huband, Maura Karr, John H. Kimbrough, Mariana Castanheira

**Affiliations:** 1 Element Iowa City (JMI Laboratories) 138461 , North Liberty, Iowa, USA; Maine Medical Center Department of Medicine, Portland, Maine, USA

**Keywords:** susceptibility testing, surrogate marker, *Aspergillus fumigatus*, isavuconazole

## Abstract

*Aspergillus fumigatus* is a common cause of pulmonary and invasive mold infections among immunocompromised hosts. Mortality in immunocompromised hosts with invasive *Aspergillus* infections (IAI) has been reported to be as high as 80%. Therefore, appropriate therapy is essential in treating IAI. Both isavuconazole and voriconazole are first-line agents in treatment guidelines for IAI, but isavuconazole has favorable properties, often leading it to be preferred over voriconazole, given the lengthy duration of treatment. It is difficult to perform mold antifungal susceptibility testing, which often requires a reference lab and several weeks to determine results. Therefore, use of surrogate markers can be helpful to infer susceptibility when testing is not possible or delayed. We performed isavuconazole and voriconazole broth microdilution susceptibility testing by the Clinical and Laboratory Standards Institute (CLSI) method on a collection of 976 non-duplicate *A. fumigatus* isolates from a global surveillance program between 2017 and 2022. We found that voriconazole and isavuconazole have a very high essential agreement within two doubling dilutions at 99.9% and a categorical agreement of 92.7% with no very major errors, one major error (0.11%), and <10% minor errors. Many of the minor errors were in the setting of voriconazole testing at a MIC of 0.5 mg/L (susceptible) but isavuconazole at 2 mg/L (intermediate). Genetic analysis of *cyp51* genes confirmed that isavuconazole and voriconazole susceptibility testing identified isolates with *cyp51A* and *cyp51B* mutations. Voriconazole can be used to predict the isavuconazole susceptibility testing result when *A. fumigatus* is tested by CLSI broth microdilution methodology.

## INTRODUCTION

*Aspergillus* spp. are ubiquitous environmental organisms that can be found in soil, water, and air (1). In people with altered lung architecture or immunocompromising conditions, *Aspergillus* spp. can cause pulmonary disease or invasive *Aspergillus* infections (IAIs) with spread to the bloodstream and other bodily sites. This organism can also cause chronic aspergillosis and allergic aspergillosis. *Aspergillus fumigatus* is the most common etiology of IAI and is reported to be the causative agent in up to 67% of cases (2). The 1-year mortality rate in immunocompromised hosts with IAI is reported to be 30%–80% (3–5). Given this high mortality rate, appropriate antifungal treatment is essential for patients with IAI.

There are currently three classes of antifungal therapeutics with activity against *Aspergillus* spp.: polyenes (amphotericin B), echinocandins, and azoles. Amphotericin B has the most reliable activity against *A. fumigatus*, but its use is limited by toxicity, and guideline recommendations are to use it only when voriconazole cannot be administered (6). Echinocandins are not recommended to be used as single-agent treatment for *A. fumigatus* infections (6). Therefore, azoles are the agents of choice for empiric and targeted therapy of *A. fumigatus*, with both voriconazole and isavuconazole recommended as first-line agents (6). Treatment duration is recommended to be a minimum of 6–12 weeks (6).

Fungal surveillance testing has shown that the rates of azole-resistant *A. fumigatus* have been increasing, most likely due to both prolonged patient exposure to azoles and the use of agricultural fungicides (7, 8). The most commonly identified mechanisms of azole resistance in *A. fumigatus* are in the *cyp51A* and *cyp51B* genes with either tandem repeats in the promoter region or single-nucleotide polymorphisms within the genes. However, 25%–50% of isolates that are resistant to azoles by Clinical and Laboratory Standards Institute (CLSI) guidelines do not have identified mutations in the *cyp51A* and *cyp51B* genes, indicating other azole resistance mechanisms are present and not routinely identified (9). Non-cyp51-mediated azole resistance mechanisms include overexpression of efflux pumps, gain-of-function mutations in transcription factors, and mutations in sterol biosynthesis components (9). Given the rising antifungal resistance rates and high mortality of IAI, it is essential to have antifungal susceptibility testing results to allow targeted treatment of infections even though routine susceptibility testing is not yet recommended by the 2016 Infectious Diseases Society of America guidelines (6).

Susceptibility testing of filamentous fungi is difficult to perform, laborious, and therefore is only done in specialized reference labs (10). Currently, there is a published breakpoint for voriconazole against *A. fumigatus* (11). A provisional breakpoint for isavuconazole has also been proposed, and epidemiologic cutoff values (ECVs) exist for isavuconazole, posaconazole, and itraconazole for *A. fumigatus* to define wild-type and non-wild-type (NWT) populations of this organism (12). Isavuconazole has advantages over voriconazole in the treatment of IAI in terms of pharmacokinetics, side-effect profile, and lack of the need for therapeutic drug monitoring (13). Given the difficulty with susceptibility testing of *A. fumigatus*, we have compared susceptibility testing results from 976 clinical isolates of *A. fumigatus* from a worldwide surveillance program against both voriconazole and isavuconazole to determine the reliability of voriconazole as a surrogate marker for predicting isavuconazole susceptibility.

## MATERIALS AND METHODS

### Organisms

A total of 976 non-duplicate *A. fumigatus* isolates were collected from clinical infections globally from 2017 to 2022 as part of the SENTRY Antimicrobial Surveillance Program. A total of 45 medical centers, including 18 in North America, 17 in Europe, and 10 in other countries, provided isolates included in this analysis. A total of 514 isolates were from Europe; 318 were from North America (44 from Canada and 274 from the United States); 128 were from the Asia–Pacific region; and 16 were from Latin America. Identification was performed using matrix-assisted laser desorption ionization time-of-flight mass spectrometry (Broker, Billerica, MA). If isolates did not score ≥1.8 by spectrometry, identification was performed using sequencing of the 28S ribosomal subunit with subsequent analysis of β-tubulin or internal spacer regions as previously described (14). Nucleotide sequences were analyzed using Lasergene software (DNASTAR, Madison, WI) and compared to reference sequences using BLAST.

### Susceptibility testing

All isolates were tested by broth microdilution according to CLSI M38M51S and as previously described (8, 11). Briefly, frozen-form microdilution panels using RPMI 1640 broth supplemented with morpholinepropane sulfonic acid buffer and 0.2% glucose were inoculated with 0.4–5.0 × 10^4^ CFU/mL of conidial suspensions, leading to a final concentration of 0.2–2.5 × 10^4^ CFU/mL. Minimal inhibitory concentrations (MICs) for azoles were read after incubation for 48 hours at 35°C. The MIC was determined as the lowest drug concentration producing visually clear wells (100% inhibition of growth). Quality control was performed according to CLSI M38M51S using *A. flavus* ATCC 204304 and *A. fumigatus* ATCC MYA-3626. Clinical breakpoints were defined for voriconazole as susceptible (S), ≤0.5 mg/L; intermediate (I), 1 mg/L; resistant (R), ≥2 mg/L; and for isavuconazole as S, ≤1 mg/L; I, 2 mg/L; R, ≥4 mg/L (11, 12).

### Genetic analysis

Mutations in the sterol 14α-demethylase-encoding gene (*cyp51A* and *cyp51B*) were analyzed for all (*n* = 44) isolates that were intermediate or resistant to both isavuconazole and voriconazole as previously described (15). Sequences were compared to the GenBank sequences available under accession numbers AAK73659.1 for *cyp51A* and AAK73660.1 for *cyp51B*. In-depth analysis of isolates with these mutations has been previously published and therefore is not performed here (8).

## RESULTS

### Antifungal susceptibility testing

**Fig 1:**
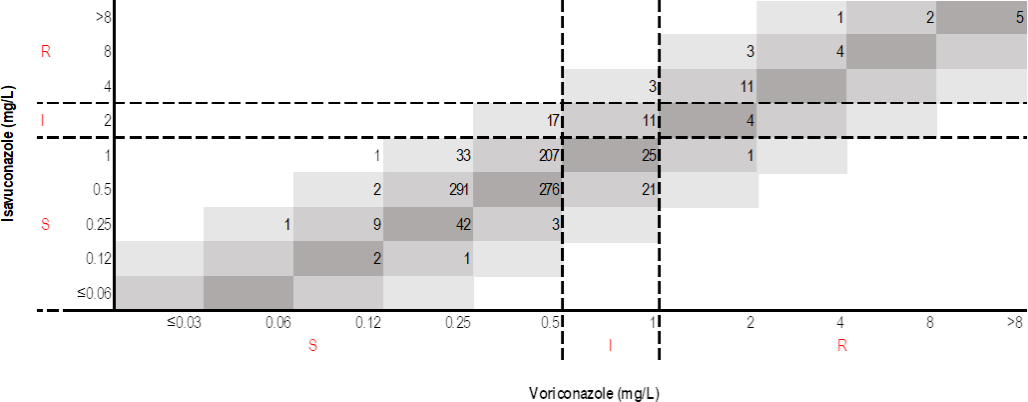
Scattergram for *Aspergillus fumigatus* isavuconazole MIC (mg/L) vs voriconazole MIC (mg/L) using CLSI MIC breakpoint interpretive criteria. S, susceptible; I, intermediate; R, resistant).

MIC values for voriconazole ranged from 0.06 to >8 mg/L, and those for isavuconazole ranged from 0.12 to >8 mg/L. Of the 976 clinical *A. fumigatus* isolates that were tested, 885 (90.7%) were susceptible to voriconazole; 60 (6.1%) were intermediate to voriconazole; and 31 (3.2%) were resistant to voriconazole. Based on the provisional breakpoints, of the 976 clinical *A. fumigatus* isolates tested, 915 (93.8%) were susceptible to isavuconazole; 32 (3.3%) were intermediate to isavuconazole; and 29 (3.0%) were resistant to isavuconazole. From the prior ECV definitions, 31 isolates were NWT to voriconazole (MIC >1 mg/L; 31 of 976, 3.2%), and 61 isolates were NWT to isavuconazole (MIC >1 mg/L; 61 of 976, 6.3%). A scattergram of the MIC distributions for isavuconazole and voriconazole is shown in Fig. 1.

Of the isolates that were susceptible to voriconazole, 868 were susceptible to isavuconazole and 17 were intermediate to isavuconazole; none were resistant to isavuconazole. Of the isolates that were intermediate to voriconazole, 46 were susceptible to isavuconazole; 11 were intermediate to isavuconazole; and 3 were resistant to isavuconazole. Of the 31 isolates that were resistant to voriconazole, 1 was susceptible to isavuconazole; 4 were intermediate to isavuconazole; and 26 were also resistant to isavuconazole.

The calculated error rates were low when voriconazole was used as a surrogate for isavuconazole susceptibility testing (Table 1). There were no very major errors (0.0%), 1 major error (0.1%), and 70 minor errors (7.2%) when using the provisional CLSI breakpoints for isavuconazole for comparison. The categorical and essential agreements were high for tested isolates (Table 2). Of the 976 isolates, 356 (36.3%) had the same MIC for isavuconazole and voriconazole. Within one doubling dilution, the essential agreement of the isavuconazole and voriconazole MICs was 93.9%, and within two doubling dilutions, the essential agreement was 99.9%. The overall categorical agreement was 92.7%.

**TABLE 1:** Error rates when voriconazole is used as surrogate for isavuconazole susceptibility testing for *A. fumigatus^a^*

Interpretation	Number of isolates	Discrepancy rates		
		Very major (%)	Major (%)	Minor (%)
R	29	0 (0.0)	N/A	3 (10.3)
I	32	N/A	N/A	21 (65.6)
S	915	N/A	1 (0.11)	46 (5.0)
Total	976	0 (0.0)	1 (0.1)	70 (7.2)

^*a*^ R, resistant; I, intermediate; S, susceptible; N/A, not applicable.

**TABLE 2:** Comparison of MIC values for isavuconazole and voriconazole against tested isolates of *A. fumigatus* with essential and categorical agreement

	Number of isolates	%
Essential agreement		
Same MIC	354	36.3
±1 dilution	915	93.8
±2 dilutions	975	99.9
Categorical agreement	905	92.7

### Genetic characterization

All 44 of the isolates that were resistant or intermediate to both isavuconazole and voriconazole had genetic analysis of *cyp51A* and *cyp51B* performed (Table 3). Of these isolates, 8 were wild-type for both Cyp51A and Cyp51B; 28 had amino acid alterations in Cyp51A; 6 had amino acid alterations in Cyp51B; and 2 had amino acid alterations in both Cyp51A and Cyp51B (Table 3). Of the isolates that were wild-type in both genes, three (37.5%) were resistant or intermediate to isavuconazole and resistant to voriconazole, while five (62.5%) were resistant or intermediate to isavuconazole and intermediate to voriconazole. The most common alteration in Cyp51A was TR_34_/L98H in 21 of 44 isolates (47.7%). The isolates with this substitution were generally resistant to both isavuconazole and voriconazole (20 of 21, 95.2%, one intermediate isolate to both). G488S in Cyp51A was found in 2 of the 44 isolates which were both resistant to isavuconazole and voriconazole (4.5%). There were single instances of TR_46_/Y121F/M172I/T289A/G448S (resistant to both drugs), F46Y/M172V/N248T/D255E/E427K (intermediate to both drugs), I242V, F46Y/M172V/E427K (intermediate to both drugs), and TR_46_/Y121F/T289A (resistant to both drugs). The most common alteration in Cyp51B was Q42L in 7 of the 44 isolates (15.9%). Isolates with this substitution were generally intermediate to both drugs (four of seven, 57.1%); one was intermediate to isavuconazole but resistant to voriconazole; one intermediate to voriconazole but resistant to isavuconazole; and one isolate was resistant to both agents but also contained a Cyp51A alteration. P383L was found in one isolate (intermediate to isavuconazole and voriconazole).

**TABLE 3:** ISA and VRC non-susceptible *A. fumigatus* isolates, including country of origin and result of *cyp* sequencing^*a*^

Isolate	State and/or country	MIC (mg/L)		Cyp alterations	
		ISA	VRC	Cyp51A	Cyp51B
1027858	New York, USA	2	1	Wild type	Q42L
1192926	Germany	2	1	Wild type	Wild type
1195605	Kansas, USA	2	1	Wild type	Wild type
1200487	New Jersey, USA	2	1	Wild type	Wild type
1177982	Turkey	2	2	Wild type	Wild type
1179127	UK	2	1	Wild type	Wild type
1201870	Michigan, USA	2	2	Wild type	Wild type
1218426	Indiana, USA	2	1	I242V	Q42L
1192458	New Jersey, USA	2	2	Wild type	Q42L
1070622	Vermont, USA	2	1	F46Y/M172V/E427K	Wild type
1072943	Czech Republic	2	1	F46Y/M172V/N248T/D255E/E427K	Wild type
1102402	New Jersey, USA	2	1	Wild type	Q42L
1169542	New Jersey, USA	2	1	Wild type	Q42L
1170842	Australia	2	1	Wild type	P383L
1122735	Italy	2	2	TR_34_/L98H	Wild type
1027901	Italy	4	2	TR_34_/L98H	Wild type
1190257	Germany	4	1	Wild type	Wild type
1151434	Indiana, USA	4	2	Wild type	Wild type
1077393	Italy	4	2	TR_34_/L98H	Wild type
1077394	Italy	4	1	TR_34_/L98H	Wild type
1077395	Italy	4	2	TR_34_/L98H	Wild type
1100802	Slovenia	4	2	TR_34_/L98H	Wild type
1174053	France	4	1	Wild type	Q42L
1075598	Belgium	4	2	TR_34_/L98H	Wild type
1122732	Italy	4	2	TR_34_/L98H	Wild type
1179124	UK	4	2	TR_34_/L98H	Wild type
1179130	UK	4	2	TR_34_/L98H	Wild type
1213028	Czech Republic	4	2	TR_34_/L98H	Wild type
1121859	UK	4	2	TR_34_/L98H	Wild type
1027905	Italy	8	2	TR_34_/L98H	Wild type
1027910	Italy	8	4	TR_34_/L98H	Wild type
1077391	Italy	8	2	TR_34_/L98H	Wild type
1190259	Germany	8	4	TR_34_/L98H	Wild type
1179131	UK	8	2	TR_34_/L98H	Wild type
1251689	France	8	4	TR_34_/L98H	Wild type
1246708	Belgium	8	4	TR_34_/L98H	Wild type
1077392	Italy	>8	>8	TR_34_/L98H	Wild type
1175751	New Zealand	>8	8	G138C	Wild type
1131576	Virginia, USA	>8	4	G448S	Wild type
1131591	Belgium	>8	>8	TR_46_/Y121F/M172I/T289A/G448S	Wild type
1201307	France	>8	>8	H147Y	Wild type
1246715	Belgium	>8	8	TR_34_/L98H	Wild type
1258509	Virginia, USA	>8	>8	G448S	Q42L
1262011	New Zealand	>8	>8	TR_46_/Y121F/T289A	Wild type

^*a*^ CLSI, Clinical and Laboratory Standards Institute; ISA, isavuconazole; MIC, minimal inhibitory concentration; VRC, voriconazole.

## DISCUSSION

Comparison of susceptibility testing for isavuconazole and voriconazole against *A. fumigatus* reveals good essential and categorical agreement among the two agents, indicating that voriconazole is an excellent surrogate marker of isavuconazole susceptibility testing. The essential agreement between testing of these two agents within two doubling dilutions was excellent at 99.9%, above the recommended threshold of ≥90% (16). The categorical agreement was 92.7%, above the ≥90% threshold recommended by CLSI (16). The very major error rate of zero was below the threshold of ≤1.5% or ≤3.0% recommended for surrogate test use (17, 18). Additionally, there was only one major error (0.11%) which is also below the recommended threshold of ≤3.0%, and there were 70 minor errors (7.17%) which are below the recommended threshold of ≤10%. The isolate representing the major error had a voriconazole MIC of 2 mg/L and an isavuconazole MIC of 1 mg/L. The minor errors that occurred were mostly (46 of 70, 65.7%) in isolates displaying an intermediate MIC value for voriconazole (MIC of 1 mg/L) but susceptible to isavuconazole (MIC of 0.5 or 1.0 mg/L). There were also 17 isolates (17 of 70, 24.3%) that tested intermediate to isavuconazole (MIC of 2 mg/L) but susceptible to voriconazole (MIC of 0.5 mg/L). There were additional minor errors with three isolates (3 of 70, 4.3%) that were intermediate to voriconazole (MIC of 1 mg/L) but resistant to isavuconazole (MIC of 4 mg/L) and four isolates (4 of 70, 5.7%) that were resistant to voriconazole (MIC of 2 mg/L) but intermediate to isavuconazole (MIC of 2 mg/L).

There may be some concern regarding the 17 isolates that tested intermediate to isavuconazole but susceptible to voriconazole, as using voriconazole as a surrogate in this case could potentially lead to clinical failure with isavuconazole. However, there is literature suggesting that isavuconazole may be successful when given in an escalated dose regimen for *A. fumigatus* isolates with an MIC of 2 mg/L, although a susceptible dose-dependent MIC is not defined by CLSI in the most recent guidelines (12, 19). Additionally, the MIC value of 2 mg/L for isavuconazole against *A. fumigatus* is defined as an area of technical uncertainty by the European Committee on Antimicrobial Susceptibility Testing (EUCAST) Subcommittee on Antifungal Susceptibility Testing due to its ambiguous interpretation (20). EUCAST recommends that at an isavuconazole MIC of 2 mg/L and a voriconazole MIC of ≤1 mg/L should be reported as susceptible, with a comment explaining the stringent susceptibility breakpoints for isavuconazole. However, direct comparisons between EUCAST and CLSI MICs are difficult to perform due to differences in testing technique, including glucose concentration in the media (less by CLSI method), different starting inoculum (higher with EUCAST), and reading MIC values by eye (CLSI) vs a spectrophotometer (EUCAST). Many of the isolates that tested intermediate to isavuconazole by CLSI testing (MIC of 2 mg/L) but susceptible to voriconazole were wild type in both *cyp51A* and *cyp51B*, which has been previously published (15). Other publications have also demonstrated that the clinical breakpoint for isavuconazole against *A. fumigatus* includes a component of the wild-type population (21). Given the lack of *cyp51* alterations among these isolates, repeat MIC testing was performed on a selection of these isolates in triplicate to determine reproducibility of the 2-mg/L value for isavuconazole (Table S1). Repeat testing showed that the 2 mg/L that was initially recorded was on the high range of the MIC distribution for these isolates, and all triplicate repeats were in the range of 0.5–1.0 mg/L for isavuconazole, reflecting their *cyp51* wild-type genotype.

In examining the *cyp51* alteration analysis of isavuconazole-intermediate or isavuconazole-resistant isolates (Table 3), interesting patterns appear. The majority of isolates that were intermediate or resistant to isavuconazole and resistant to voriconazole (27 of 30, 90.0%) were found to have c*yp51* alterations. There were two isolates with MICs of 2 mg/L to both isavuconazole and voriconazole and one isolate with MICs of 4 mg/L to isavuconazole and 2 mg/L to voriconazole that were wild-type for both *cyp51A* and *cyp51B*. These isolates warrant further exploration to understand their mechanisms of resistance. We did not evaluate efflux pump expression; upregulation may be the underlying etiology, as prior work has shown that upregulation of the Cdr1B efflux system can lead to azole resistance in *A. fumigatus* (22). As previously described, the substitutions found in *cyp51* genes leading to azole resistance vary based on isolate source location (8). In Europe, of the non-wild-type isolates, the most common alterations were in the promoter region of Cyp51A (TR_34_/L98H and TR_46_/Y121F/M172I/T289A/G448S, 22/25, 88%) (8). The azole resistance associated with mutations in the promoter region of the *cyp51A* gene is due to increased protein expression and attributed to the use of agricultural fungicides, which are more common in Europe than the United States (23). The MICs for isolates with these promoter alterations varied from 2 to >8 mg/L for isavuconazole and from 1 to >8 mg/L for voriconazole, spanning the intermediate and resistant susceptibility ranges for both drugs. None of the organisms with promoter alterations were susceptible to isavuconazole or voriconazole by MIC testing to either agent. There was also an isolate with high-level isavuconazole and voriconazole resistance (MIC >8 mg/L for both) and with two Cyp51A substitutions in combination with the TR_46_ promoter substitution (TR_46_/Y121F/T289A). In contrast, single amino acid substitutions at G54, G138, M220, and G448 more commonly occur after exposure to prior triazole therapy (24). These single amino acid substitutions were found in one isolate in Europe (H147Y), in four isolates from the United States, and in one from New Zealand. The isolate with G138C in Cyp51A was highly resistant to both isavuconazole and voriconazole with MIC values of >8 and 8 mg/L, respectively. There were two isolates with a G448S alteration in Cyp51A, and both were also highly resistant to isavuconazole (MIC of >8 mg/L) and voriconazole (MICs of 4 and >8 mg/L). The isolate with H147Y was also highly resistant to both azoles with MICs of >8 mg/L for both agents. This alteration is not as frequently reported in the literature, and its implication in azole resistance is unclear (25). The isolate with a I242V alteration in Cyp51A in combination with Q42L in Cyp51B also had low-level azole resistance with MICs of 2 mg/L for isavuconazole and 1 mg/L for voriconazole. The Cyp51A I242V substitution has been previously identified as common among itraconazole-resistant *A. fumigatus* isolates in the United States (26). Future work on all amino acid substitutions identified in this collection of azole-intermediate or azole-resistant isolates is needed to better understand the individual contribution of each alteration to the phenotypic susceptibility pattern.

Alterations in Cyp51B were mainly found in isolates from the United States (six of eight, 75.0%) but were also found in single isolates from Australia and France. Overall, Cyp51B amino acid changes were less common than amino acid changes in Cyp51A. The most commonly identified Cyp51B alteration in our collection, Cyp51B Q42L, has been previously recognized in isavuconazole–non-wild-type *A. fumigatus* (15). Our collection of isolates shows a higher proportion of organisms with this Cyp51B polymorphism than a prior study, when it was present in 3 of 35 non-wild-type isolates (8.6%) as compared to our collection, where it is in 7 of 44 isavuconazole intermediate or resistant isolates (15.9%). In our collection, isolates with Q42L in Cyp51B had isavuconazole MICs ranging from 2 to 4 mg/L with this substitution in isolation and from 2 to >8 mg/L when in combination with other alterations. Voriconazole MICs with Q42L were from 1 to 2 mg/L when the substitution was alone and from 1 to >8 mg/L when in combination with Cyp51A alterations. Further analysis is needed to understand if this alteration is increasing in prevalence and if there are any exposures that may be leading to this substitution, including why it seems to be more common in the United States than in other continents. Our surveillance collection is not curated to perform this analysis, as the isolates do not have patient exposure details or medical histories included.

We identified one other Cyp51B alteration in our collection, P383L, which has been previously described (27). The isolate with this alteration had intermediate susceptibility to both isavuconazole and voriconazole with MIC values of 2 and 1 mg/L, respectively. This alteration has not been characterized as it relates to azole susceptibility and is an important topic for future work. Like with the identified Cyp51A polymorphisms, the alterations in Cyp51B identified here require further characterization to understand their contribution to phenotypic resistance patterns in *Aspergillus fumigatus*.

Analysis of a collection of *A. fumigatus* from a global surveillance program indicates that voriconazole has excellent parameters to be used as a surrogate for isavuconazole susceptibility in situations where isavuconazole susceptibility testing is not feasible. There were no very major errors, only one major error, and high essential and categorical agreement.

## Data Availability

Data are available at BioProject ID PRJNA1186192.

## SUPPLEMENTAL MATERIAL

Table S1 jcm.01207-24-s0001.docx MIC replicates for wild-type *Aspergillus* isolates with discrepancies in voriconazole and isavuconazole susceptibilities.

ASM does not own the copyrights to Supplemental Material that may be linked to, or accessed through, an article. The authors have granted ASM a non-exclusive, world-wide license to publish the Supplemental Material files. Please contact the corresponding author directly for reuse.
