# Supplementary material for: Use of voriconazole to predict susceptibility and resistance to isavuconazole for Aspergillus fumigatus using CLSI methods and interpretive criteria
Source: J Clin Microbiol. 2024 Dec 20;63(2):e01207-24. doi: 10.1128/jcm.01207-24 (PMC11837495; doi:10.1128/jcm.01207-24)
Supplement: Table S1 — MIC replicates for wild-type Aspergillus isolates with discrepancies in voriconazole and isavuconazole susceptibilities. [file jcm.01207-24-s0001.docx]

| **Supplemental Table 1: MIC replicates of wildtype *Aspergillus* isolates with isavuconazole resistant and voriconazole-susceptible initial MIC results (replicate 1)** | | | | | | | | |
| --- | --- | --- | --- | --- | --- | --- | --- | --- |
|  | **Compound / replicate tested (MIC mg/L)** | | | | | | | |
| **collection number** | **ISA/1** | **VRC/1** | **ISA/2** | **VRC/2** | **ISA/3** | **VRC/3** | **ISA/4** | **VRC/4** |
| 1046494 | 2 | 0.5 | 0.5 | 0.5 | 0.5 | 0.5 | 1 | 0.5 |
| 1046949 | 2 | 0.5 | 0.5 | 0.5 | 0.5 | 0.5 | 0.5 | 0.5 |
| 1047452 | 2 | 0.5 | 0.5 | 0.5 | 0.5 | 0.5 | 0.5 | 0.5 |
| 1051070 | 2 | 0.5 | 1 | 0.5 | 1 | 1 | 0.5 | 0.5 |
| 1053208 | 2 | 0.5 | 1 | 1 | 1 | 0.5 | 1 | 0.5 |
| 1053211 | 2 | 0.5 | 0.5 | 0.5 | 1 | 0.5 | 1 | 0.5 |
| 1065544 | 2 | 0.5 | 0.5 | 0.5 | 0.5 | 0.5 | 0.5 | 0.5 |
| 1070650 | 2 | 0.5 | 0.5 | 0.5 | 0.5 | 0.5 | 0.5 | 0.5 |
| 1072952 | 2 | 0.5 | 0.5 | 0.5 | 0.5 | 0.5 | 0.5 | 0.5 |
| 1072958 | 2 | 0.5 | 0.5 | 0.5 | 0.5 | 0.5 | 0.5 | 0.5 |
| MIC, minimal inhibitory concentration; ISA, isavuconazole; R, resistant; VRC, voriconazole; S, susceptible | | | | | | | | |
